# Patient Satisfaction with Telemedicine in Adults with Diabetes: A Systematic Review

**DOI:** 10.3390/healthcare10091677

**Published:** 2022-09-02

**Authors:** Hidetaka Hamasaki

**Affiliations:** Hamasaki Clinic, 2-21-4 Kagoshima, Kagoshima 890-0046, Japan; h-hamasaki@umin.ac.jp; Tel.: +81-99-2503535; Fax: +81-99-2501470

**Keywords:** patient satisfaction, telemedicine, information technology, digital health, type 1 diabetes, type 2 diabetes

## Abstract

Patient satisfaction assessment is essential for improving the quality of healthcare. Diabetes management using telemedicine technology is promising in the 21st century. However, the number of randomised controlled trials (RCTs) examining the effect of telemedicine on satisfaction in patients with diabetes is limited. This systematic review aimed to summarise the current evidence on patient satisfaction with telemedicine in adults with diabetes and discuss related issues and future directions of telemedicine in patients with diabetes. The author systematically searched PubMed/MEDLINE, Embase and The Cochrane Library, and a total of six RCTs were eligible for this review. Patient satisfaction with telemedicine was as high as conventional face-to-face care; however, telemedicine appeared not to significantly increase patient satisfaction compared with conventional face-to-face care in the included studies. Significant heterogeneity was noted between the studies, including participants’ age, study duration, the method of assessing patient satisfaction and types of telemedicine. Further studies are required to provide firm evidence to healthcare providers who are willing to use telemedicine in diabetes management. Telemedicine technology has been advancing and is a key tool in providing high-quality healthcare to patients with diabetes in the 21st century.

## 1. Introduction

Information technology has rapidly advanced since the Internet was developed and became widely used in the 1990s [1]. In healthcare, information technology has been expanding in applying electronic medical records [2], performing big data analytics [3] and utilising artificial intelligence for the diagnosis and treatment of various diseases [4], which is changing the conventional medical practice. Diabetes is a chronic disease that is significantly influenced by diet, physical activity, behaviour and medication adherence in daily life; therefore, interactive communication between healthcare providers and patients via telemedicine technology is suitable for managing patients with diabetes [5]. Indeed, the use of telemedicine was effective for glycaemic control with a mean difference of haemoglobin A1c (HbA1c) of −0.415% (95% confidence interval [CI], −0.482% to −0.348%) in patients with type 2 diabetes [6] and −0.18% (95% CI, −0.04% to −0.33%) in patients with type 1 diabetes [7], and cost-effective for diabetic retinopathy screening [8]. Moreover, the implementation of telemedicine using a high-speed network system and wearable devices was an effective measure for patient care and was accepted by both clinicians and patients with high satisfaction during the coronavirus disease 2019 (COVID-19) pandemic [9,10]. Telemedicine is a promising approach for improving the quality of healthcare [11] as well as health outcomes of patients [12] and reducing operational costs of healthcare services [8] in the management of diabetes; thus, this technology will be essential to provide healthcare effectively and safely to patients with diabetes in the 21st century. The next pandemic could occur sooner than we think, and in-person healthcare may not be allowed in such a circumstance. Conversely, the effect of telemedicine on satisfaction in patients with diabetes is not fully investigated. Kruse et al. [13] examined the association between telemedicine and patient satisfaction by analysing 44 published articles and found that patient satisfaction was associated with some factors of telemedicine, including outcomes, ease of use, communication and travel time. However, the study design of the included studies was not limited to randomised controlled trials (RCTs), and only four studies were noted in which study participants were patients with diabetes. Pascoe [14] stated that patient satisfaction assessment is critical for understanding the function of the healthcare system. Moreover, patients with high satisfaction are likely to have high medication adherence, leading to improved health outcomes [15].

Hence, assessing patient satisfaction with telemedicine in diabetes management is crucial. The author developed the following research question: In patients with diabetes (P), what is the effect of telemedicine (I) on patient satisfaction (O) compared with conventional care (C)? This study aims to investigate the effect of telemedicine on patient satisfaction in patients with diabetes.

## 2. Materials and Methods

This systematic review was conducted following the Preferred Reporting Items for Systematic Reviews and Meta-Analyses guidelines [16] (Supplementary File).

### 2.1. Search Strategy

The author searched PubMed/MEDLINE, Embase and The Cochrane Library from their inception to June 2022. The author used Medical Subject Headings (MeSH) terms ‘telemedicine’ AND ‘diabetes mellitus’ AND ‘patient satisfaction’ for the systematic search. Based on the National Institute of Health’s definition of telemedicine [17] and the study by Sood et al. [18], telemedicine is defined as the use of communication technologies for delivery of healthcare services at a distance, which involves virtual visits between physicians and patients and remote monitoring. Telemedicine is broadly defined as the use of various communication modalities [17]. There could be some studies that refer to a specific communication modality alone. Therefore, the author also used the search terms ‘virtual visit’ OR ‘digital health’ OR ‘remote monitoring’ OR ‘mHealth’ OR ‘eHealth’ as a substitute for the MeSH term ‘telemedicine’. The author included articles in English that were published in peer-reviewed journals.

### 2.2. Inclusion and Exclusion Criteria

The articles had to meet the following criteria: (1) the study design had to be RCT, (2) the study population had to be adults with types 2 or 1 diabetes, (3) the intervention had to be telemedicine implementation or telemedicine in addition to conventional in-person care and (4) the study had to report a pre- and post-satisfaction score. Reviews, observational studies, case reports, editorials, letters, conference papers and study protocols were excluded. Qualitative studies without quantitative analysis for patient satisfaction were excluded. Studies that reported patient satisfaction in the intervention group alone were also excluded.

### 2.3. Study Quality Assessment

The revised Cochrane risk-of-bias tool for randomised trials was used for quality assessment in the included studies [19]. The assessment was categorised into one of the following three levels: ‘high risk of bias’, ‘some concerns’ and ‘low risk of bias’ based on risk-of-bias tools [20].

## 3. Results

### 3.1. Study Selection

**Figure 1:**
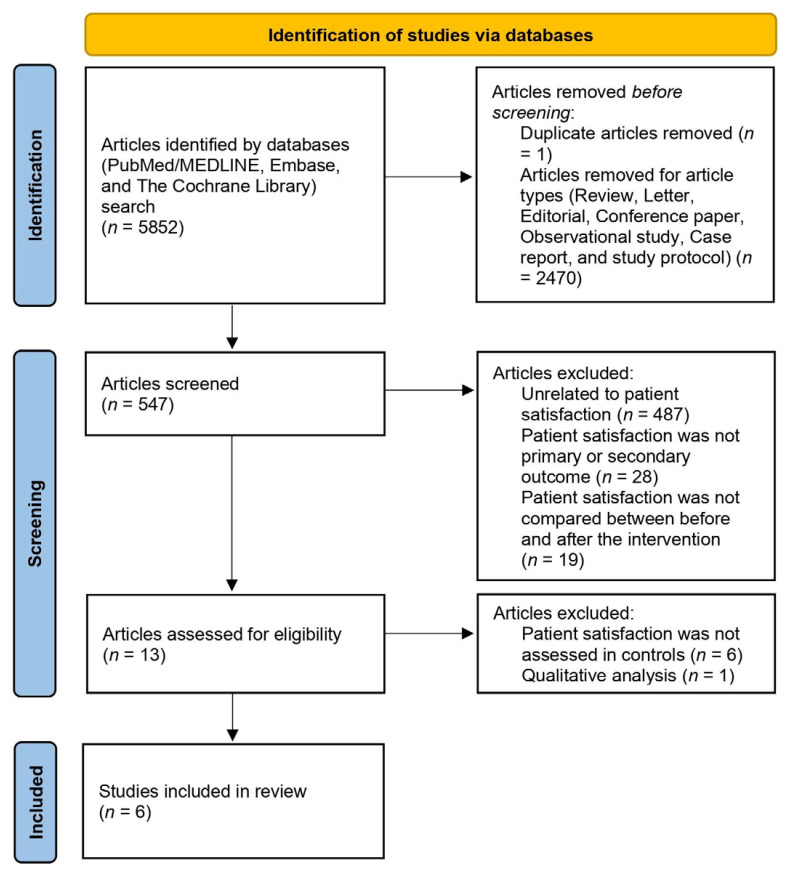
PRISMA flow diagram.

The systematic literature search yielded 5852 articles. Of these, 547 RCTs were identified. Moreover, 60 studies that investigated the effect of telemedicine on patient satisfaction in patients with diabetes were fully reviewed to determine their relevance. Six articles were eligible for this systematic review. The flow of the systematic search process is depicted in Figure 1.

### 3.2. Study Characteristics

The characteristics of the included studies are summarised in Table 1.

Izquierdo et al. [21] investigated the hypothesis that diabetes education via telemedicine was as effective as in-person for the management of patients with diabetes. Patients in the intervention group had three diabetes education visits via teleconferencing, whereas patients in the control group had one-on-one in-person visits. The first visit included 1-h consultations with a diabetes specialist nurse and dietitian. The second and third visits were 30-min consultations at 4–6 and 8–12 weeks, respectively. Physicians in charge were unaware whether the patient was allocated to the intervention or control group. Of the study participants, 88% completed the three diabetes education visits and their changes in glycaemic control and satisfaction were analysed. Although glycaemic control measured by HbA1c levels and patient satisfaction in patients who received telemedicine were not superior to those in patients who received conventional in-person care, telemedicine was as effective as in-person care for treating diabetes, and patient satisfaction was similarly high in both groups. The treatment satisfaction measured by the Diabetes Treatment Satisfaction Questionnaire (DTSQ) (a maximum total score of 36) increased from 22.8 ± 8.6 to 31.3 ± 4.2 in the intervention group (*p* < 0.001). Furthermore, the authors administered the Telemedicine Patient Satisfaction Survey [22] in the intervention group at the 3-month visit and found that overall satisfaction was relatively high (4.3 ± 1.3 on a scale of 1–5) and 84% of the participants wanted to continue telemedicine. The authors concluded that diabetes education via telemedicine would provide an opportunity for patients with diabetes who could not acquire high-quality care due to underserved areas and help motivate and empower them to improve health-related behaviours.

Yaron et al. [23] examined the effectiveness, safety, acceptability and cost-effectiveness of telemedicine in patients with type 1 diabetes using insulin pumps. Patients in the intervention group downloaded blood glucose data from their insulin pumps once a month and transferred the information to the clinic using a web application. Physicians reviewed the data and sent recommendations immediately on adjusting insulin doses and support messages to maintain and improve motivation. Additionally, study participants had face-to-face visits once at 6-month intervals, totalling three visits during the study period. Conversely, patients in the control group had face-to-face visits only at 3-month intervals, totalling five visits during the study period. Patient satisfaction with the diabetes treatment was high in both groups: the satisfaction scores were 1.8 ± 1.24 and 1.4 ± 1.3 in the intervention and control groups, respectively (scores ranged from +3 ‘much more satisfied now’ to −3 ‘much less satisfied now’). Furthermore, patients in the intervention group were more satisfied with continuing the telemedicine than those who received standard care (2.1 ± 1.21 vs. 1.4 ± 1.35, *p* = 0.04). Although the improvement in glycaemic control was not statistically significant, the frequency of hypo- and hyperglycaemic events did not differ between groups, and the direct and indirect total costs of care were reduced by 24% and 22%, respectively, in patients receiving telemedicine; hence, the telemedicine approach was highly satisfactory, safe and cost-effective in the management of patients with type 1 diabetes.

Cho et al. [24] considered the barrier that older adults could not use manual Internet-based telemedicine successfully and investigated the efficacy and feasibility of an Internet-integrated device, which automatically uploads patient data for diabetes management. The blood glucose and blood pressure data of the study participants were automatically uploaded to the online server using the health gateway device. Study participants could also communicate with the healthcare providers about their weight, diet, exercise, hypoglycaemic events and any other questions through the device and received personal recommendations every week (for the first 3 months) or every other week (for the last 3 months). The total DTSQ score was not significantly increased from 25.0 ± 6.3 at baseline to 27.9 ± 6.48 at the end of the study in the intervention group as well as that of the control group (25.9 ± 5.9–26.7 ± 5.8). However, the total DTSQ score was significantly higher in the intervention group than that in the control group at the end of the study (*p* < 0.05). Additionally, HbA1c levels and waist circumference were decreased more in the intervention group than those in the control group. No significant difference in adverse events between groups was reported. The authors did not discuss patient satisfaction in the discussion section; however, high patient satisfaction was the result of improved glycaemic control rather than telemedicine itself.

Kirwan et al. [25] investigated the effectiveness of a freely available smartphone application on the management of patients with type 1 diabetes. Patients in the intervention group were instructed to use a smartphone application that allows users to enter data regarding diabetes self-management, such as diet, physical activity, blood glucose, insulin doses and other medications in addition to usual care (face-to-face visit every 3 months). They also received personalised feedback from a certified diabetes educator to improve diabetes management at least once a week during the first six months. The mean score of ‘Satisfaction‘ in the Diabetes Quality of Life (DQOL) questionnaire was increased both in the intervention and control groups (3.20 ± 0.66–3.42 ± 0.68 and 3.09 ± 0.55–3.29 ± 0.65, respectively); however, this finding was not statistically significant, and no difference between groups was observed. Glycaemic control measured by HbA1c levels was significantly improved in the intervention group compared with that in the control group. This result suggests that patient satisfaction was not associated with improving glycaemic control. The authors pointed out that the level of engagement of study participants in telemedicine studies seemed to be underreported [26]. Even if evidence was noted of its effectiveness in clinical study settings, telemedicine without in-person communication between patients and healthcare providers may not frequently be practical for diabetes self-management in the real world.

Ruiz de Adana et al. [27] examined the impact of telemedicine on glycaemic control; health-related quality of life, including patient satisfaction with treatment; physicians’ satisfaction in patients with type 1 diabetes. Patients in the control group were followed up by face-to-face visits every 3 months, whereas those in the intervention group performed the second visit remotely using an Internet-based telemedicine system that helps patients make decisions on diabetes management. Patients in the intervention group could check the information on self-monitored blood glucose values, insulin doses, carbohydrate consumption, physical activity and other health-related data. HbA1c levels, the number of hypoglycaemia episodes and indices of glycaemic variability did not differ between groups at the end of the study. The satisfaction score with treatment increased in the control group and slightly decreased in the intervention group; however, no significant difference between groups was observed at the end of the study. Conversely, physicians’ satisfaction score with the telemedicine system was 6.28 (on a scale of 0–10). The authors stated that the clinical efficacy and safety of the telemedicine system were similar to conventional face-to-face visits. Thus, some face-to-face visits could be replaced by telemedicine, which would result in improving access to healthcare in cases with low medical resources.

Sood et al. [28] conducted a cluster-RCT to compare HbA1c, blood pressure, lipid profile and patient satisfaction measured by DTSQ between patients who had telemedicine consultations via videoconference once a week and those who had usual face-to-face consultations at the diabetes clinic. HbA1c levels were decreased in both groups; however, no statistically significant difference between groups was noted. The DTSQ score changed from 24.1 ± 0.8 to 23.8 ± 7.3 in the intervention group and from 23.8 ± 7.3 to 24.0 ± 7.7 in the control group, respectively. The change in the DTSQ score did not differ between groups. However, more patients in the intervention group were highly satisfied with the visit than those in the control group (61.2% vs. 40.8%, *p* = 0.004). Furthermore, more patients in the intervention group answered that specialists understood the patient’s situation (97.0% vs. 88.4%, *p* = 0.009) and were comfortable with the number of providers during the visit (83.0% vs. 72.3%, *p* < 0.001), and fewer patients in the intervention group answered on the disagreement of the benefit of the visits (2.4% vs. 14.1%, *p* < 0.001) than those in the control group. More than 99% of the participants agreed that telemedicine improved accessibility to medical care. These results indicated that telemedicine was as effective as usual care for managing diabetes and patient experience with telemedicine was highly appreciated.

**Table 1:** Summary of included studies.

Reference	Country	Study Design	Study Period	Subjects	Study Outcomes	Intervention Control Telemedicine Tool	Results (Patient Satisfaction)
Izquierdo et al. (2003) [21]	USA	Randomised, controlled, parallel-group trial	3 months	56 adults with diabetes, 10 dropouts Intervention group (thirteen men and nine women): Age: 61.37 ± 8.95 years, BMI: 31.34 ± 6.20 kg/m^2^, HbA1c: 8.33 ± 1.63% Control group (eight men and sixteen women): Age: 53.95 ± 10.08 years, BMI: 35.95 ± 9.22 kg/m^2^, HbA1c: 8.68 ± 2.17%	Primary: HbA1c Secondary: Weight, BMI, PAID scale, DQOL score, ADS score, DTSQ score, Treatment satisfaction	Telemedicine (videoconference)/in-person Real-time one-on-one teleconferencing using a private ISDN line.	Patient satisfaction ↑ (DTSQ score: 22.8 ± 8.6 to 31.3 ± 4.2, *p* < 0.001 in the intervention group; 23.8 ± 7.9 to 29.1 ± 5.3, *p* < 0.001 in the control group). No difference in satisfaction scores between groups. No interaction of group by time.
Yaron et al. (2019) [23]	Israel	Randomised, controlled, parallel-group trial	12 months	74 patients with type 1 diabetes on insulin pumps, seven dropouts Intervention group (19 men and 12 women): Age: 43 ± 11 years, BMI: 26.6 ± 4.6 kg/m^2^, HbA1c: 7.59 ± 0.82% Control group (13 men and 23 women): Age: 45 ± 14 years, BMI: 25.5 ± 3.8 kg/m^2^, HbA1c: 7.93 ± 0.6%	Primary: HbA1c Secondary: QoL score (ADDQoL score), DTSQ score, changes in total hypoglycaemic events (blood glucose ≤ 70 mg/dL) and hyperglycaemic events (blood glucose ≥ 300 mg/dL), cost-effectiveness of telemedicine	Telemedicine (Carelink Pro Software)/Face-to-face visit Data from the insulin pump and glucometer are transmitted to the clinic by Carelink Pro Software. Physicians review the data and document the recommendations.	Patient satisfaction ↑ DTSQ score of Q8 ‘willingness to continue’ was higher in the intervention group than that in the control group (2.1 vs. 1.4, *p* = 0.04). QoL →
Cho et al. (2017) [24]	South Korea	Randomised, controlled, parallel-group trial	6 months	484 patients with type 2 diabetes Intervention group (152 men and 88 women): Age: 53.4 ± 8.7 years, BMI: 25.5 ± 3.2 kg/m^2^, HbA1c: 7.81 ± 0.66% Control group (155 men and 89 women): Age: 52.9 ± 9.2 years, BMI: 25.6 ± 3.4 kg/m^2^, HbA1c: 7.86 ± 0.89%	Primary: HbA1c, glycaemic control Secondary: Anthropometric and biochemical parameters, SF-12 score, DTSQ score, adverse events of telemedicine	Internet-based communication (HiCare)/Outpatient clinic visit Patients upload the glucose and blood pressure data automatically to the online server using the device. Patients can see recommendation messages from the medical team on the device.	Patient satisfaction ↑ (DTSQ score: 25.0 ± 6.3 to 27.9 ± 6.48, *NS* in the intervention group; 25.9 ± 5.9 to 26.7 ± 5.8, *NS* in the control group). DTSQ score was significantly higher in the intervention group than that in the control group 6 months after the intervention (27.9 ± 6.48 vs. 26.7 ± 5.8, *p* < 0.05). QoL →
Kirwan et al. (2013) [25]	Australia	Randomised, controlled, parallel-group trial	9 months	72 adults with type 1 diabetes, 19 dropouts Intervention group (19 men and 17 women): Age: 35.97 ± 10.67 years, BMI: No description, HbA1c: 9.08 ± 1.18% Control group (nine men and twenty-sevem women): Age: 34.42 ± 10.26 years, BMI: No description, HbA1c: 8.47 ± 0.86%	Primary: HbA1c Secondary: DES-SF score, SDSCA score, DQOL score	Smartphone application (Glucose Buddy)/Usual care Freely available iPhone application that allows patients to manually enter diet, physical activity, blood glucose levels, insulin dosages and other medications. The application data were reviewed by a certified diabetes educator via a web interface.	Patient satisfaction → (DQOL score ‘Satisfaction’: 3.20 ± 0.66 to 3.42 ± 0.68, *NS* in the intervention group; 3.09 ± 0.55 to 3.29 ± 0.65, *NS* in the control group). No difference in satisfaction scores between groups. No interaction of group by time. No change in either group in relation to self-efficacy, self-care activities and QoL.
Ruiz de Adana et al. (2020) [27]	Spain	Randomised, controlled, parallel-group trial	6 months	388 patients with type 1 diabetes, 58 dropouts Intervention group (90 men and 73 women): Age: 33.78 ± 9.77 years, BMI: 26.0 ± 4.6 kg/m^2^, HbA1c: 66 participants < 7% Control group (94 men and 73 women): Age: 36.22 ± 10.78 years, BMI: 26.0 ± 4.6 kg/m^2^, HbA1c: 68 participants < 7%	Primary: Mean change of HbA1c Secondary: Mean blood glucose, glycaemic variability, DQOL score, DDS score, FH-15 score	Telemedicine (Diabetic) + face-to-face visit/Face-to-face visit Internet-based telemedicine system (web- and mobile-based) designed for monitoring patients with diabetes. Patients can download their self-monitored blood glucose data and include information regarding carbohydrate consumption, physical activity, insulin dosages and other health data. Physicians can review the data and evaluate metabolic control statistics or treatment reports.	Patient satisfaction → (DQOL score ‘Satisfaction’: 72.0 ± 12.4 to 71.7 ± 14.8, NS in the intervention group; 67.8 ± 16.1 to 69.5 ± 16.3, NS in the control group). The satisfaction score (satisfaction) was higher in the intervention group than that in the control group (72.0 vs. 67.8 *p* < 0.05) at the first visit; however, no difference in satisfaction between groups was observed at the end of study.
Sood et al. (2018) [28]	USA	Cluster-randomised, controlled, trial	18 months	282 patients with diabetes Intervention group (one hundred ninety-eight men and one woman): Age: 61.6 ± 9.4 years, BMI: No description, HbA1c: 10.0 ± 1.6% Control group (83 men): Age: 61.1 ± 10.0 years, BMI: No description, HbA1c: 9.4 ± 2.1%	Primary: HbA1c Secondary: Medication use, blood pressure, low-density lipoprotein cholesterol, high-density lipoprotein cholesterol, triglycerides, DTSQ score	Telemedicine (videoconference)/Usual care Telemedicine consultation via videoconference at the community-based outpatient clinic accompanied by one healthcare provider. The medical team interviews and advises the patient to manage diabetes effectively.	Patient satisfaction → (DTSQ score: 24.1 ± 8.0 to 23.8 ±7.3, *NS* in the intervention group; 23.8 ± 7.3 to 24.0 ± 7.7, *NS* in the control group). No difference in satisfaction scores between groups. However, the proportion of patients who were very satisfied with the consultation was higher in the intervention group than that in the control group (61.2% vs. 40.8%, *p* = 0.004).

↑ Increase, → no change, *BMI* body mass index, *HbA1c* haemoglobin A1c, *PAID* Problem Areas in Diabetes, *DQOL* Diabetes Quality of Life, *ADS* Appraisal of Diabetes Scale, *DTSQ* Diabetes Treatment Satisfaction Questionnaire, *ISDN* integrated services digital network, *QoL* quality of life, *ADDQoL* Audit of Diabetes Quality of Life, *NS* not significant, *SF-12* 12-item Short Form Survey, *DES-SF* Diabetes Empowerment Scale-Short Form, *SDSCA* Summary of Diabetes Self-Care Activities, *DDS* Diabetes Distress Scale, *FH-15* Fear of Hypoglycaemia scale.

Table 2 and Table 3 visually summarise the relationship of patient satisfaction with glycaemic control (changes in HbA1c levels) (Table 2) and telemedicine modalities (Table 3). There was no significant relationship of patient satisfaction with glycaemic control and a specific telemedicine modality.

### 3.3. Study Quality

**Figure 2:**
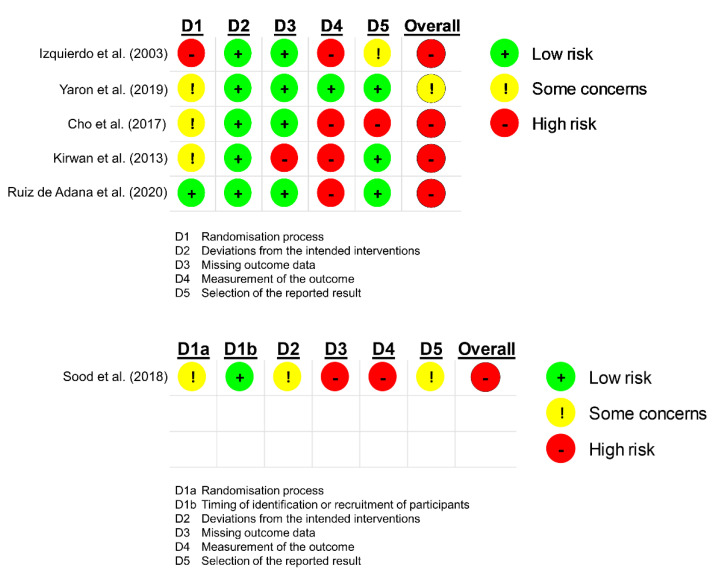
Risk of bias in the included studies. This figure was automatically made by RoB2 tool: https://www.riskofbias.info/welcome/rob-2-0-tool/current-version-of-rob-2 (accessed on 1 January 2022).

The overall risk of bias was ‘high‘ except for the study by Yaron et al. [23] (Figure 2).

Most studies involved some concerns in the random sequence process, and the studies by Izquierdo et al. [21], Kirwan et al. [25] and Sood et al. [28] had some differences in participant characteristics between the intervention and control groups at baseline. The most critical factor for the high risk of bias in the included studies is the open-label design, except for the study by Yaron et al. [23] (single-blinded). Therefore, the outcome measurements may have been influenced by researcher biases.

## 4. Discussion

This systematic review demonstrated that patient satisfaction was high in patients receiving telemedicine. However, most of the included studies reported no significant difference in patient satisfaction between the telemedicine and usual (face-to-face) care groups. The study by Cho et al. [24] showed that telemedicine significantly increased patient satisfaction compared with face-to-face visits; however, this result may have been influenced by the course of treatment for diabetes because the study participants were not blinded and were aware about how their glycaemic control changed during the study period. In contrast, Ruiz de Adana et al. [27] indicated that patient satisfaction could decrease with the implementation of telemedicine; however, patients in the intervention group used telemedicine in only one of three visits, which suggests that the effect of telemedicine alone on patient satisfaction was not sufficiently evaluated.

Significant heterogeneity was observed between the included studies, such as participants’ age, study duration and the method of assessing patient satisfaction. However, the telemedicine tool used in previous studies varied. The article by Izquierdo et al. [21] was published in 2003; thus, data transmission speed via the Internet was limited. The study participants communicated with each other using Integrated Services Digital Network (128 Kb/s) in real-time, which is much slower than the current Fifth Generation Mobile Communication System (5G), whose theoretical data transmission speed is ~10–30 Gb/s [29]. No specific telemedicine device/application was used in this study. Similarly, Yaron et al. [23] and Sood et al. [28] did not use a specific device/application (Yaron et al. used software that enabled reviewing patients’ data from the insulin pump and glucometer). In contrast, Cho et al. [26] used a health gateway system (HiCare) with Internet-based communication, Kirwan et al. [25] used a smartphone application and Ruiz de Adana et al. [27] used a remote monitoring system that allowed access to patients’ data from smartphones. Each system or application has different functionality and usability for the management of diabetes; hence, it may be challenging to assess patient satisfaction with ‘telemedicine‘ as a whole concept.

The sufficient validity and reliability of the patient satisfaction questionnaire are essential for patient satisfaction surveys. For example, the Telemedicine Patient Satisfaction Survey [22] used in the study by Izquierdo et al. [21] is an established approach to assess patient satisfaction, which was extensively tested in previous studies [30,31]; however, according to Hajesmaeel-Gohari and Bahaadinbeigy [32], this questionnaire is not frequently used these days.

In contrast, DTSQ is widely used for evaluating patient satisfaction with the treatment of diabetes [33]. Four of six studies in this systematic review used DTSQ to assess patient satisfaction. DTSQ consists of eight question items that are rated on a seven-point Likert scale (ranging from zero to six): Q1 ‘How satisfied are you with your current treatment?’, Q2 ‘How often have you felt that your blood sugars have been unacceptably high recently?’, Q3 ‘How often have you felt that your blood sugars have been unacceptably low recently?’, Q4 ‘How convenient have you been finding your treatment to be recently?’, Q5 ‘How flexible have you been finding your treatment to be recently?’, Q6 ‘How satisfied are you with your understanding of your diabetes?’, Q7 ‘Would you recommend this form of treatment to someone else with your kind of diabetes?’ and Q8 ‘How satisfied would you be to continue with your present form of treatment?’ [34]. Considering that this questionnaire aims to assess patient satisfaction in terms of diabetes treatment, that is, diet, exercise, type and dose of oral hypoglycaemic agents and insulin injections, it should be noted that satisfaction with the healthcare delivery system may not be adequately evaluated. Conversely, the other two studies used the DQOL questionnaire to evaluate patient satisfaction. The DQOL questionnaire has the following four sections: ‘Satisfaction‘, ‘Impact‘, ‘Worry: Social/Vocational‘ and ‘Worry: Diabetes Related’. The Satisfaction section consists of the following 15 items that are rated on a five-point Likert scale (ranging from one to five): Q1 ‘How satisfied are you with the amount of time it takes to manage your diabetes?’, Q2 ‘How satisfied are you with the amount of time you spend getting checkups?’, Q3 ‘How satisfied are you with the time it takes to determine your sugar level?’, Q4 ‘How satisfied are you with your current treatment?’, Q5 ‘How satisfied are you with the flexibility you have in your diet?’, Q6 ‘How satisfied are you with the burden your diabetes is placing on your family?’, Q7 ‘How satisfied are you with your knowledge about your diabetes?’, Q8 ‘How satisfied are you with your sleep?’, Q9 ‘How satisfied are you with your social relationships and friendships?’, Q10 ‘How satisfied are you with your sex life?’, Q11 ‘How satisfied are you with your work, school, and household activities?’, Q12 ‘How satisfied are you with the appearance of your body?’, Q13 ‘How satisfied are you with the time you spend exercising?’, Q14 ‘How satisfied are you with your leisure time?’ and Q15 ‘How satisfied are you with life in general?’ [35]. The Satisfaction section of the DQOL questionnaire is designed to assess satisfaction with not diabetes management but a patient’s whole life; however, Q1 and Q2 are important question items because they are related to the time it takes to manage diabetes and receive health checkups. Recently, AshaRani et al. [36] reported that the advantages of telemedicine included saving time, convenience and cost, and the most satisfactory telemedicine service was booking appointments. The results of this study suggest that patients expect improved access to healthcare through the implementation of telemedicine. Therefore, the questionnaire should include question items regarding accessibility, such as waiting time, appointment booking system and healthcare costs, to appropriately assess changes in patient satisfaction. The most frequently used telemedicine-specific questionnaire is the Telehealth Usability Questionnaire, which includes the question items regarding time travelling to healthcare organisations and access to healthcare services [32]; however, no question item on costs was noted. Telemedicine-specific questionnaires cannot be used to assess satisfaction in patients with conventional face-to-face care. A new comprehensive questionnaire should be developed to accurately assess patient satisfaction with telemedicine and validly compare patient satisfaction between patients receiving telemedicine and face-to-face care.

Interestingly, compared with patients without diabetes, those with diabetes prefer face-to-face visits over telemedicine [36]. Furthermore, most (75.7%) patients with diabetes seem to be not ready for telemedicine compared with those without diabetes (44.4%) owing to the following disadvantages of telemedicine: (1) requirement of digital literacy (94.3%), (2) difficulty in building good interpersonal relationships between clinicians and patients (81.0%), (3) not being credible (64.2%) and (4) not helpful for patients’ health conditions (62.3%) [36]. Diabetes is a chronic disease, which makes continuity of care crucial. Patients who had a good interpersonal relationship with primary care physicians and high continuity of care had reduced mortality (odds ratio [OR] = 0.59; 95% CI, 0.50–0.70) and a lower risk of hospitalisation (OR = 0.82; 95% CI, 0.75–0.90) in addition to achieving better diabetes control [37]. Patient-centred care and interpersonal behaviours by healthcare providers could improve diabetes patients’ trust and engagement in their management and the healthcare system [38]. Patient satisfaction is built based on individual subjective perception [39]; thus, objective assessment and the elimination of confounding factors are difficult. Moreover, the most crucial factor for building patient satisfaction is the interpersonal relationship between each patient and the healthcare provider [40]; therefore, who provides care to the patient may have more impact than what the healthcare provider uses for patient care. However, all the included studies did not mention differences in healthcare providers between telemedicine and in-person groups. The interpersonal relationship between healthcare providers and patients should be considered to truly assess the effect of telemedicine on patient satisfaction in patients with diabetes. Furthermore, patients’ social relationships with their families, peers and healthcare providers significantly impact diabetes management across the life span [41]. Therefore, patients with diabetes may value a face-to-face relationship in their self-management.

One of the significant barriers to the implementation of telemedicine is the lack of digital literacy [42]. Particularly, older adults with lower income who are not familiar with digital devices have been found to experience difficulty in using telemedicine [43,44]. A systematic review concluded that older adults were satisfied with telemedicine during the COVID-19 pandemic; however, technical difficulties concerning the telemedicine delivery system were major barriers [45]. The average age of participants in Izquierdo et al. [21] and Sood et al. [28] was >60 years old, which may have affected patient satisfaction with telemedicine. However, most of the participants in the included studies were relatively young (30–50 years old), and the effect of telemedicine on patient satisfaction in older patients with diabetes was unknown. Considering that ageing is rapidly progressing worldwide, further studies investigating the effect of telemedicine on patient experience in older patients with diabetes are warranted.

Information technology has achieved a remarkable breakthrough. However, the author recognises the vital need for further technological advancements in healthcare to provide effective and efficient treatment to the increasing number of older patients worldwide. For example, 5G networks will be able to transfer a significantly higher volume of data than now, allowing healthcare providers to perform more real teleconsultation and telesurgery [46]. Moreover, the Internet of Things technology will assist older adults living alone with maintaining or improving activities of daily life [47]. Such information technology advancement in healthcare ensures connecting older adults and their families with healthcare resources and may improve physical and mental well-being [48], which contributes to establishing an integrated healthcare system in ageing communities.

Furthermore, the author has mentioned the issue of the study design in the included studies. In principle, implementing double-blind RCTs with subjective endpoints, such as patient satisfaction, is impossible. Study participants can realise which group they were allocated and how they were treated. However, at least the outcome assessor should be blinded (single-blind), as in the study by Yaron et al. [23].

This systematic review had some limitations. First, it was conducted by a single author; hence, biases in study selection and study quality assessment may exist. A systematic review with meta-analysis should be performed in the future with the inclusion of an adequate number of RCTs. Second, all of the included studies were not designed to assess patient satisfaction as a primary outcome. Further studies investigating the effect of telemedicine on patient satisfaction as a primary outcome are needed.

In conclusion, patient satisfaction was high in patients receiving telemedicine; however, the difference in patient satisfaction between telemedicine and face-to-face care was not significant. Considerable heterogeneities between studies were noted, including age and telemedicine delivery tools. Moreover, the effect of telemedicine on patient satisfaction as a primary outcome in older patients who are not ready for telemedicine due to a lack of digital literacy was not examined. Telemedicine should be more studied and developed in anticipation of the next pandemic or coming superageing societies. Telemedicine that establishes good interpersonal relationships between patients and healthcare providers and increases patient satisfaction is a key tool to providing high-quality healthcare to patients with diabetes in the 21st century.

## Figures and Tables

**Table 2:** The relationship between patient satisfaction and glycaemic control.

Study	Glycaemic Control (HbA1c)	Patient Satisfaction
Izquierdo et al. [21] Cho et al. [24]	↓	↑
Yaron et al. [23]	→	↑
Kirwan et al. [25]	↓	→
Ruiz de Adana et al. [27] Sood et al. [28]	→	→

↑ Increase, → no change, ↓ decrease, *HbA1c* haemoglobin A1c.

**Table 3:** The relationship between patient satisfaction and telemedicine modalities.

Study	Telemedicine Modalities	Patient Satisfaction
Izquierdo et al. [21] Sood et al. [28]	Virtual visit	↑ →
Cho et al. [24] Yaron et al. [23] Kirwan et al. [25]	Remote monitoring	↑ ↑ →
Ruiz de Adana et al. [27]	Virtual visit + Remote monitoring	→

↑ Increase, → no change, ↓ decrease.

## Data Availability

The data that support the findings of this study are available from the corresponding author upon reasonable request.

## Supplementary Materials

The following supporting information can be downloaded at: https://www.mdpi.com/article/10.3390/healthcare10091677/s1, PRISMA 2020 checklist.
